# Goodness Better Than Greatness

**Published:** 1871-02

**Authors:** 


GOODNESS BETTER THAN GREATNESS.—We all naturally desire "great things" for our children. But too often we forget how much better goodness is than greatness. "Water will always find its level," so of the numbers which go to make up our communities. Lay a substantial foundation for the character in noble, manly, generous principles, and your boy will not fail to succeed in life. Guide and counsel him wisely, but do not attempt to force him into a calling for which his taste and talents totally unfit him.—*Working Farmer.*

